# Combined Photothermal and Photodynamic Therapy for Cancer Treatment Using a Multifunctional Graphene Oxide

**DOI:** 10.3390/pharmaceutics14071365

**Published:** 2022-06-28

**Authors:** Shi Guo, Zhengmei Song, Ding-Kun Ji, Giacomo Reina, Jean-Daniel Fauny, Yuta Nishina, Cécilia Ménard-Moyon, Alberto Bianco

**Affiliations:** 1 CNRS, Immunology, Immunopathology and Therapeutic Chemistry, UPR3572, University of Strasbourg, ISIS, 67000 Strasbourg, France; guoshi54@hotmail.com (S.G.); zm.song@ibmc-cnrs.unistra.fr (Z.S.); jdk1234@163.com (D.-K.J.); reina.giacomo.25u@st.kyoto-u.ac.jp (G.R.); jd.fauny@ibmc-cnrs.unistra.fr (J.-D.F.); c.menard@ibmc-cnrs.unistra.fr (C.M.-M.); 2 Graduate School of Natural Science and Technology, Okayama University, Tsushimanaka, Kita-ku, Okayama 700-8530, Japan; nisina-y@cc.okayama-u.ac.jp; 3 Research Core for Interdisciplinary Sciences, Okayama University, Tsushimanaka, Kita-ku, Okayama 700-8530, Japan

**Keywords:** Carbon nanomaterials, phototherapy, targeting, chlorin e6, macrophages

## Abstract

Graphene oxide (GO) is one of the most studied nanomaterials in many fields, including the biomedical field. Most of the nanomaterials developed for drug delivery and phototherapies are based on noncovalent approaches that lead to an unspecific release of physisorbed molecules in complex biological environments. Therefore, preparing covalently functionalized GO using straightforward and versatile methods is highly valuable. Phototherapies, including photothermal therapy (PTT) and photodynamic therapy (PDT), have shown great potential as effective therapeutic approaches against cancer. To overcome the limits of a single method, the combination of PTT and PDT can lead to a combined effect with a higher therapeutic efficiency. In this work, we prepare a folic acid (FA) and chlorin e6 (Ce6) double-functionalized GO for combined targeted PTT/PDT. This conjugate can penetrate rapidly into cancer cells and macrophages. A combined effect of PTT and PDT is observed, leading to a higher killing efficiency toward different types of cells involved in cancer and other diseases. Our work provides a simple protocol to prepare multifunctional platforms for the treatment of various diseases.

## 1. Introduction

Phototherapies, including photothermal therapy (PTT) and photodynamic therapy (PDT), have attracted tremendous scientific interest for the treatment of various diseases, especially cancer, due to their unique advantages, such as minimal invasiveness, limited side effects, negligible drug resistance and high efficiency [1,2,3,4]. PTT requires photothermal materials to convert the energy of absorbed photons into heat, resulting in a local hyperthermia, which leads to the death of cells by triggering necrosis or apoptosis [5,6]. In the case of PDT, cancer cells are instead killed by the reactive oxygen species (ROS) generated by photosensitizers, which transfer energy to the adequate oxygen contents in tissues under light irradiation [3,7,8].

Although many studies have proven the promising potential of individual PDT or PTT in disease treatment [1,9], there is a growing interest in combining PDT and PTT in a single system for a combined therapy aiming to improve the therapeutic efficacy [10,11,12]. In the case of the single photodynamic treatment, an important therapeutic problem is that the depletion of tissue oxygen would exacerbate the local hypoxia, decreasing the efficiency of reactive oxygen species (ROS) generation due to the insufficiency of molecular oxygen [13,14,15]. To overcome the hypoxia effect, PDT could be combined with PTT to improve the delivery of oxygen since the localized hyperthermia can enhance the blood circulation to the tumor site, leading to an increased oxygen content [16]. In the case of PTT, a laser with high intensity (normally higher than 0.5 W per cm^2^) is usually required to ensure sufficient hyperthermia [17]. However, intense light irradiation might induce side effects to healthy tissues penetrated by light [18]. By combining with PDT, PTT can be applied using a lower laser power. Milder hyperthermia can sensitize cells to the influence of other treatments by improving the cellular uptake of drugs and reducing tissue hypoxia [10,19,20]. Thus, the combination of PDT and PTT could lead to better therapeutic efficiency with minimal side effects.

To date, most of the photothermal agents are based on inorganic materials, such as metal oxides and gold nanoparticles [12,19,21]. However, their low biodegradability and potential long-term cytotoxicity severely limit their future translation in the clinics [11]. Graphene oxide (GO) is one of the alternative nanomaterials for photothermal therapy thanks to its biocompatibility and biodegradability [22], high colloidal stability [23] and good photothermal conversion efficiency [17,24]. GO is the oxidized form of graphene, consisting of a hexagonal ring-based carbon network with abundant oxygen-containing functional groups, which provide GO with abundant reactive sites for the loading of functional molecules through an epoxide ring opening [25], esterification [26] or other covalent reactions [27,28]. The high surface area associated with the presence of oxygenated groups allows the loading of molecules, including photosensitizers for PDT [29]. GO was also used in photothermal therapies against cancer [30,31,32]. By derivatizing with targeting ligands and photosensitizers, GO can be used for both PTT and PDT to target cancer cells for a higher therapeutic effect [33].

Herein, we designed and synthesized a multifunctionalized GO conjugate for cancer treatment, combining PTT and PDT. A double-functionalized GO containing folic acid (FA) and chlorin e6 (Ce6) was first prepared. FA has a high affinity toward the folate receptor (FR) in cancer cells. The FR is overexpressed in several human tumors, including breast, ovary, lung, kidney cancer and tumor-associated macrophages, while its expression in normal tissues is low [34,35]. Ce6 was selected as a photosensitizer for the photodynamic treatment. This molecule can generate singlet oxygen under laser irradiation at 660 nm [36]. Both FA and Ce6 were covalently grafted onto GO through a polyethylene glycol (PEG) linker. We demonstrated that the GO–FA/Ce6 conjugate had good photothermal properties and the ability to generate ROS triggered by Ce6 irradiation. This system was able to target the MCF-7 cancer cell line via ligand–receptor interactions with a good killing efficiency by PTT and PDT alone. After combining PTT and PDT, a high combined effect was observed, leading to a better therapeutic outcome.

Moreover, we also extended the use of this nanomaterial against macrophages, which are potential targets for cancer therapy [37,38] and autoimmune diseases [39,40,41]. It is reported that FR is highly overexpressed in tumor-associated macrophages [42] and inflammation-activated macrophages, such as in rheumatoid arthritis joint, making them a potential target for anti-inflammatory therapy [43]. For example, FR on RAW 264.7 cells could specifically recognize FA-conjugated nanomaterials leading to a receptor-mediated endocytosis [44]. In this study, RAW 264.7 macrophages resulted in being more sensitive to PTT than PDT. On the other hand, a combined effect was observed on macrophages with a higher killing efficiency when PTT and PDT were subsequently applied.

This work provides a flexible method for a mild functionalization of the GO surface, allowing the design of conjugates with a variety of therapeutic molecules and targeting ligands to tackle and treat various types of diseases. Our robust and controlled functionalization strategy leads to a higher loading efficiency than the noncovalent method with similar or better therapeutic efficiency using a lower amount of photosensitizer, which is crucial for minimizing side effects during cancer therapy.

## 2. Materials and Methods

### 2.1. Synthesis of the Building Blocks, Precursors and Final GO Conjugates

#### 2.1.1. Preparation of GO Suspensions

GO material was prepared by the modified Hummers’ method [45]. Briefly, graphite was added to H_2_SO_4_ with the slow addition of KMnO_4_ at 10 °C while stirring at 200 rpm. The mixture was kept at 35 °C for 2 h and quenched with water, while temperature was kept under 50 °C. H_2_O_2_ was slowly added, with continuous stirring, for 30 min at ambient temperature. The reaction mixture was purified by centrifugation.

All GO suspensions were prepared by sonication in a water bath (20 W, 40 kHz) with a controlled temperature between 20 °C and 30 °C.

#### 2.1.2. Synthesis of Boc-PEG_10_-FA

441 mg (1 mmol) of folic acid and 200 μL of triethylamine (TEA) were dissolved in 5 mL of DMSO by continuous stirring at room temperature for 10 min under argon. The solution was cooled to 4 °C, and EDC∙HCl (191 mg, 1 mmol) and NHS (143 mg, 1.2 mmol) were added, and the mixture was stirred for 24 h. The mixture was dropped to cold DCM (200 mL), and the precipitate was filtered over a polytetrafluoroethylene (PTFE) Millipore^®^ membrane with 0.1 µm pore size, obtaining a yellowish solid, subsequently used without further purification. The recovered solid was washed with DCM and dissolved in 5 mL DMF under argon. The solution was cooled to 4 °C, and 644 mg (1 mmol) of Boc-PEG_10_-NH_2_ was added. After stirring for 24 h, the mixture was dropped into cold diethyl ether with continuous stirring. The diethyl ether was removed by filtration, and the precipitate was washed again with cold diethyl ether to afford a yellowish oily product. The crude product was purified by silica gel column using acetone/DCM/EtOH/H_2_O/TEA = 3/2/1/0.7/0.3 as eluant, to first eliminate the side products and then changing to acetone/DCM/EtOH/H_2_O/TEA = 2/2/2/0.7/0.3 to obtain the desired product. After drying in vacuum, a yellowish solid corresponding to Boc-PEG_10_–FA (310 mg, 28.6% yield) was obtained. LC-MS (ESI): m/z calculated for C_48_H_77_N_9_O_18_ 1067.54, found 1068.08 [M+H]^+^. HPLC: Rt = 9.8 min over 20 min of gradient: 0–100% of A and 100–0% of B in 20 min at 1.2 mL∙min^−1^ flow rate (A = H_2_O + 0.1% TFA, eluent B = MeCN + 0.08% TFA).

#### 2.1.3. Synthesis of FA–PEG_10_-NH_2_

FA–PEG_10_-NH_2_ was prepared by mixing 300 mg of Boc-PEG_10_–FA with 5 mL TFA stirring for 2 h at room temperature. TFA was removed under reduced pressure. The crude product was dissolved in ethyl acetate and washed with NaHCO_3_ solution and water. The FA–PEG_10_-NH_2_ was obtained under reduced pressure and directly used without further purification.

#### 2.1.4. Preparation of GO–FA

To a suspension of GO (80 mg) in 16 mL of water, 230 mg of Boc-PEG_10_-NH_2_ and 230 mg of FA–PEG_10_-NH_2_ were added to the solution and bath sonicated for 10 min. The mixture was then stirred at room temperature for 3 days and filtered over a PTFE membrane with 0.1 µm pore size. The solid on the membrane was collected, dispersed in water, sonicated in a water bath and filtered again. This sequence was repeated 3 times with water. The solid was finally dispersed in water. The suspension was dialyzed in water for 3 days and lyophilized to obtain GO–FA.

#### 2.1.5. Preparation of GO–FA–CTR

The preparation of GO–FA–CTR was similar to the preparation of GO–FA. To a suspension of GO (20 mg) in 5 mL of water, 125 mg of Boc-PEG_10_-NH_2_ were added to the solution and bath sonicated for 10 min. The mixture was then stirred at room temperature for 3 days and filtered over a PTFE filter membrane with 0.1 µm pore size. The solid on the membrane was collected and washed with water 3 times. The solid was finally dispersed in water, and the suspension was dialyzed in water for 3 days and lyophilized to obtain GO–FA–CTR.

#### 2.1.6. Preparation of GO–FA/Ce6

The GO–FA was first deprotected using 4 M HCl in 1,4-dioxane. To a suspension of GO–FA (60 mg) in 30 mL of 1,4-dioxane sonicated for 10 min, 30 mL of 4 M HCl in 1,4-dioxane was added. The suspension was sonicated for 5 min and stirred for 17 h and filtered over a PTFE filter membrane with 0.1 µm pore size. The solid on the membrane was collected and washed with water 3 times. The Boc-deprotected GO–FA was dried under vacuum. Ce6 (80 mg) was dissolved in 1 mL of DMF and 20 μL of TEA and stirred in an ice bath for 10 min. EDC·HCl (28.2 mg) was added to the solution, which was stirred for 1 h in the ice bath. Then, 23.1 mg of NHS were added to the mixture, and the solution was stirred for 17 h at 4 °C. The mixture was dropped to a suspension of 20 mg Boc-deprotected GO–FA in 9 mL of DMF, which was stirred for 17 h. The mixture was filtered over a PTFE filter membrane with 0.1 µm pore size. The solid on the membrane was collected and washed with DMF and water 3 times each. The solid was finally dispersed in water, and the suspension was dialyzed in water for 3 days and lyophilized to obtain GO–FA/Ce6.

#### 2.1.7. Preparation of GO–FA/Ce6–CTR

GO–FA/Ce6–CTR was obtained following the protocol for the preparation of GO–FA/Ce6 but directly using GO–FA for the reaction without Boc-deprotection. Ce6 (40 mg) was dissolved in 1 mL of DMF and 10 μL of TEA, and the solution was stirred in an ice bath for 10 min. EDC·HCl (14.1 mg) was added to the solution, which was stirred for 1 h in the ice bath. Then, 11.5 mg of NHS were added to the mixture, and the solution was stirred for 17 h at 4 °C. The mixture was dropped into a suspension of 10 mg GO–FA in 4 mL of DMF and stirred for 17 h. The mixture was filtered over a PTFE filter membrane with 0.1 µm pore size. The solid on the membrane was collected and washed with DMF and water 3 times each. The solid was finally dispersed in water, and the suspension was dialyzed in water for 3 days and lyophilized to obtain GO–FA/Ce6–CTR.

## 3. Results and Discussion

### 3.1. Synthesis and Characterization of FA/Ce6 Double-Functionalized GO

**Scheme 1:**
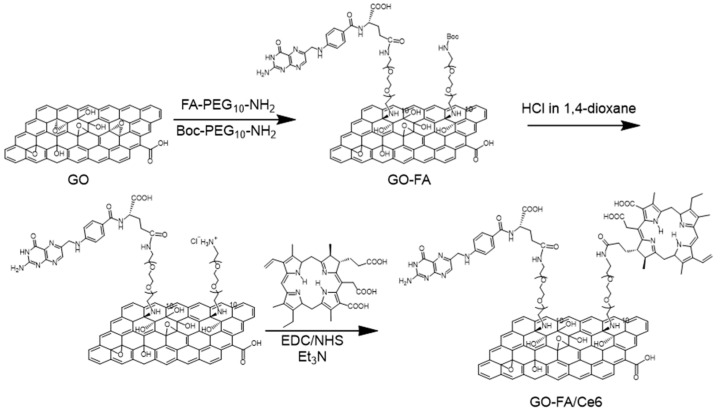
Synthesis of GO–FA/Ce6 conjugate (the three carboxyl groups of Ce6 can participate in the amidation reaction, but for clarity, we illustrated the modification of only one of them).

GO was prepared by the modified Hummers’ method [45,46], with an average lateral size of about 1 μm. FA and Ce6 were covalently grafted onto the GO surface in three steps (Scheme 1). The FA derivative, namely FA–PEG_10_-NH_2_, was initially prepared by an amidation reaction between FA and a mono-Boc-protected PEG diamine chain (Boc-PEG_10_-NH_2_), followed by the Boc deprotection in an acidic solution. GO was then derivatized using an equimolar mixture of the two free amine-terminated PEG ligands, namely Boc–PEG_10_-NH_2_ and FA–PEG_10_-NH_2_, through the nucleophilic opening of the epoxides. The Boc group on GO was then deprotected, and Ce6 was conjugated to the free amines through an amidation reaction using the coupling reagents 1-ethyl-3-(3-dimethylaminopropyl)carbodiimide hydrochloride (EDC·HCl) and *N*-hydroxysuccinimide (NHS).

**Figure 1:**
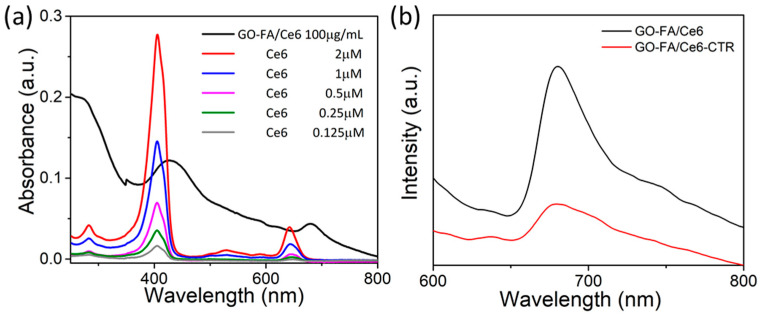
(**a**) UV-Vis spectra of GO–FA/Ce6 and Ce6 alone at different concentrations in water and (**b**) fluorescence spectra of GO–FA/Ce6 and GO–FA/Ce6–CTR dispersed in water at 10 μg/mL (λ_ex_ = 420 nm).

UV-Vis spectroscopy was employed to prove the presence of FA and Ce6 on the GO surface and to evaluate the loading of Ce6 (Figure 1a). The characteristic absorbance of FA centered at 290 nm was easily identified in GO–FA. In addition, GO–FA/Ce6 also presents two new characteristic peaks at 420 nm (Soret band) and at 681 nm (Q band) belonging to Ce6 with a red shift compared to Ce6 alone, due to the strong interaction between Ce6 and GO [36,47]. Although the UV-Vis spectrum of Ce6 changed after binding to GO, we could still estimate the loading efficiency using the standard curve of the Ce6 solution by measuring the Soret band absorption around 420 nm. The amount of Ce6 loaded on the double-functional GO was estimated to 3 μmol·g^−1^ (Figure S1). Due to the covalent functionalization, the loading efficiency of Ce6 was much higher than physisorption by mixing GO–FA with the same amount of Ce6 used for the amidation reaction (GO–FA/Ce6–CTR, see Figure S2 for details). Compared to the covalent reaction, GO–Ce6 also shows a shift of the Soret band and Q band. For a better calculation of Ce6 adsorbed onto GO–FA/Ce6–CTR, we doubled the amount of the material for the UV-Vis spectrum. The loading efficiency was estimated as 0.9 μmol·g^−1^, which is much lower than GO–FA/Ce6. The GO–FA/Ce6 showed a stronger fluorescence emission at 650 nm upon excitation at 420 nm, compared to the signal of Ce6 in the control (GO–FA/Ce6–CTR), proving again that a higher loading efficiency of Ce6 was achieved through amidation when the same starting amount of Ce6 was used for preparing the double-functionalized materials (Figure 1b). The thermogravimetric analysis also confirmed the successful derivatization of GO (Figure S3). Compared to the pristine GO, a higher weight loss was observed in the region between 200 and 400 °C, likely due to the contribution of the thermal degradation of the PEG chains covalently bound to GO [48].

**Figure 2:**
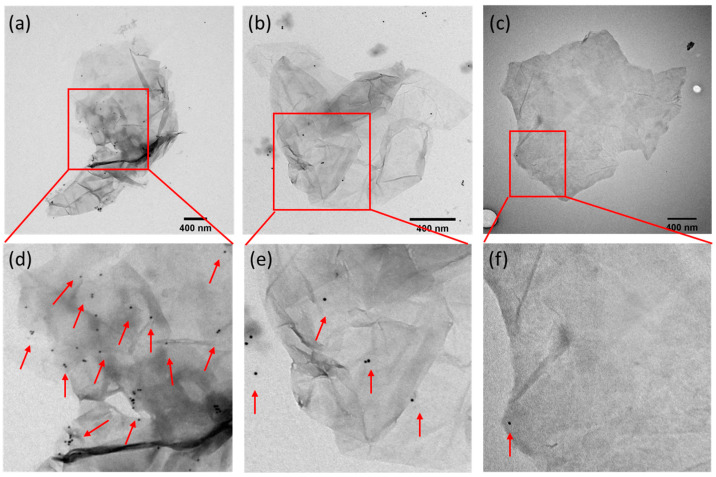
TEM images (**a**–**c**) and magnified images (**d**), 3× magnification of (**a**); (**e**), 3× magnification for (**b**); (**f**), 4× magnification for (**c**) illustrating the immunostaining of (**a**,**d**) GO–FA, (**b**,**e**) GO–FA–CTR using an antiFA antibody and a secondary antibody and (**c**,**f**) GO–FA using the nonspecific antibody hRANK-M331 and a secondary antibody. The gold nanoparticles are pointed out with red arrows.

To further prove the presence of FA, an immunostaining was applied on GO–FA. For this analysis, two antibodies were used. Copper grids deposited with GO–FA were incubated with an antiFA antibody and a secondary antibody coupled to 10 nm gold nanoparticles, which allowed the identification of the antibody by TEM. Many gold nanoparticles were observed on the GO surface, revealing that a large quantity of FA was grafted onto GO (Figure 2a,d). To verify that the gold nanoparticles were not nonspecifically adsorbed onto GO, a control sample without FA was prepared by reacting GO with only Boc-PEG_10_-NH_2_, giving GO–FA–CTR (see Supporting Information for details). The immunostaining of GO–FA–CTR showed only rare gold nanoparticles present on the GO (Figure 2b,e), confirming that the secondary antibody was bound to the antiFA antibody onto GO–FA sheets rather than simply nonspecifically physisorbed. Moreover, another control primary antibody, the hRANK-M331 antibody (a mouse IgG1 monoclonal antibody against the human protein RANK-M331), was used followed by the incubation with the secondary antibody bearing gold nanoparticles. The hRANK-M331 antibody does not have affinity for FA, but it can be recognized by the secondary antibody. TEM showed no gold nanoparticles on GO–FA incubated with hRANK-M331 and the secondary antibody, proving again the specificity of the immunostaining using the antiFA antibody (Figure 2c,f).

### 3.2. Photothermal Property and ROS Generation Capacity

**Figure 3:**
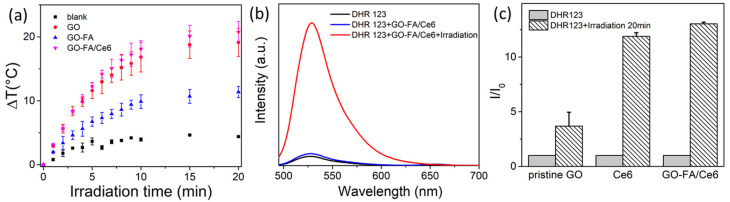
(**a**) Temperature increases in aqueous dispersions of GO, GO–FA and GO–FA/Ce6 at different time points under 808 nm irradiation, (**b**) DHR123 test for ROS generation by GO–FA/Ce6 under 660 nm irradiation and (**c**) normalized DHR123 fluorescence for ROS generation by GO, Ce6 and GO–FA/Ce6.

Following the characterization of the conjugate, we investigated the photothermal effects induced by a near-infrared laser irradiation at 808 nm of aqueous dispersions of GO, GO–FA and GO–FA/Ce6 (Figure 3a). Thanks to its broad absorbance profile, GO and its derivatives are considered good photothermal materials to treat various diseases [49]. After applying a laser at a power of 2 W·cm^−2^, the temperature increased immediately at the concentration of 25 μg·mL^−1^ of the different materials tested. The temperature increased by 17 °C for GO and GO–FA/Ce6 within 10 min, which could induce irreversible damage on cells [6,17], while GO–FA showed a lower increase in temperature probably because of its low water dispersibility likely due to the poorly soluble FA and Boc protecting group. The removal of the Boc moiety and the presence of the additional carboxyl groups of Ce6 on GO–FA/Ce6 improved the water dispersibility of the final conjugate. The thermal stability of the conjugates was verified by irradiation at 808 nm for 10 min, confirming that the structure of Ce6 is not affected (data not shown).

The generation of ROS by GO–FA/Ce6 was subsequently evaluated by fluorescence spectroscopy using dihydrorhodamine 123 (DHR123). This dye is not fluorescent, but after oxidation by ROS, it is transformed into fluorescent rhodamine 123, with a high red emission [50,51]. As shown in Figure 3b, the fluorescence emission of DHR123 was almost negligible in water or after the addition of 10 μg·mL^−1^ of GO–FA/Ce6, proving that the conjugate did not change the fluorescence of the dye. After applying the irradiation using a 660 nm laser at the low power of 0.2 W·cm^−2^ for 10 min, a significant increase in the fluorescence intensity was observed, due to the generation of ROS by the photosensitizer. In the meantime, DHR123 alone was stable when exposed to 660 nm laser as the fluorescence signal did not increase during the irradiation. The ROS generated by Ce6 alone were also detected by DHR123. Surprisingly, the generation of ROS by GO–FA/Ce6 was slightly higher, although not significantly different than Ce6 alone at the same concentration (Figure 3c). The higher ROS production of GO–FA/Ce6 could be due to the additional ROS generated by GO under exposure to laser light [52,53].

### 3.3. Cellular Uptake in MCF-7 Cells

**Figure 4:**
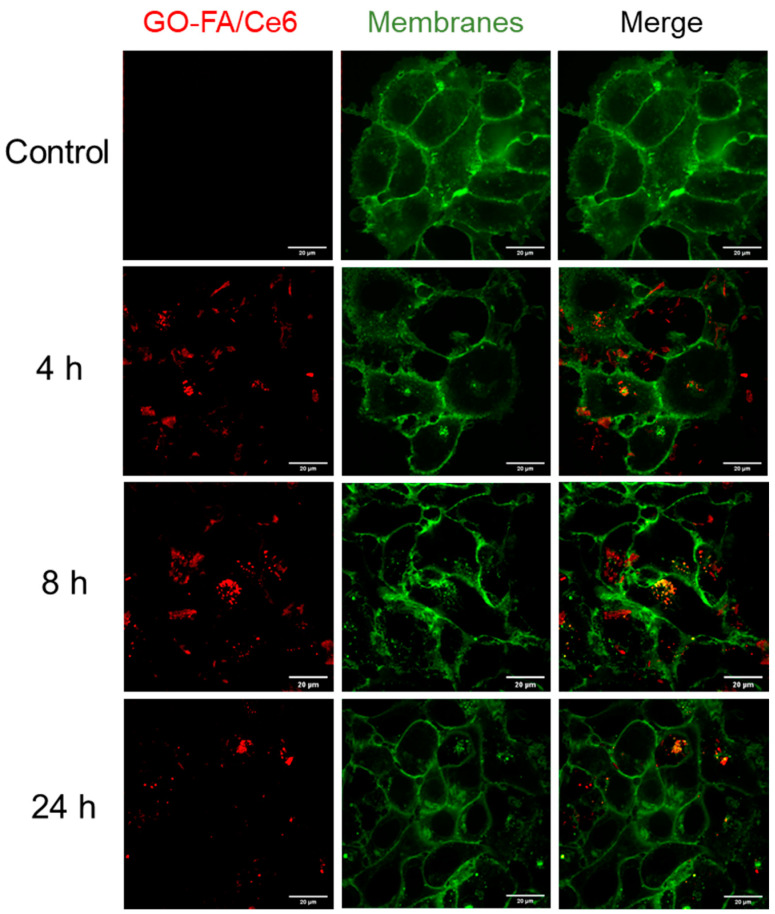
Confocal microscopy images showing the cellular uptake of GO–FA/Ce6 by MCF-7 cells at different time points. The untreated MCF-7 cells were used as control. In green, membranes stained with CellMask (excitation: 488 nm, emission: 500–550 nm); in red, GO–FA/Ce6.

In order to exploit the photothermal and photodynamic properties for therapeutic applications, we evaluated the cellular uptake of GO–FA/Ce6 by MCF-7 cells using confocal microscopy, by tracking the fluorescence of Ce6 on GO at different time points (0, 4, 8 and 24 h) (Figure 4). CellMask green was applied for better identification of the cell membranes. GO–FA/Ce6 was already internalized by the MCF-7 cells within 4 h, indicating a quick internalization of the nanomaterial due to endocytosis mediated by FR on MCF-7 cells. A higher accumulation of GO–FA/Ce6 was observed in the cytoplasmic compartments 8 h after the treatment with the nanomaterial. On the basis of these results, we believe that it is preferential to perform the photothermal and photodynamic experiments within 4 h to 24 h incubation using the double-functional GO–FA/Ce6. We chose 4 h and 8 h incubation for the subsequent experiments due to the higher fluorescence of Ce6.

**Figure 5:**
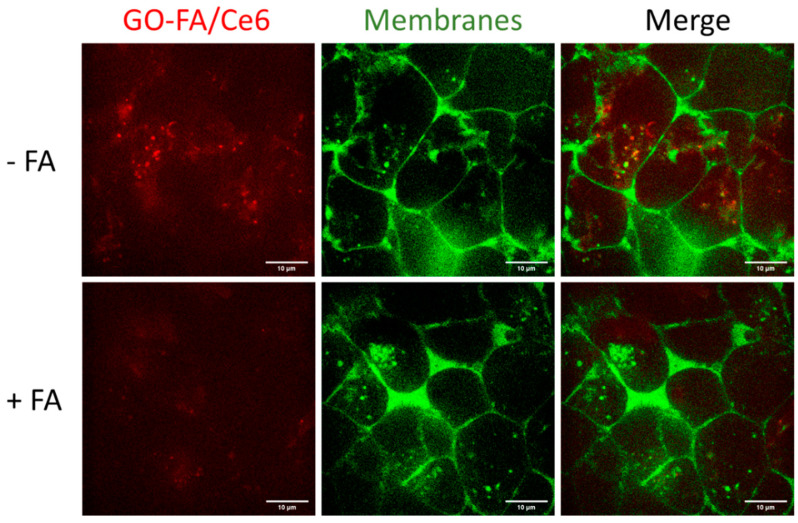
Confocal microscopy images of MCF-7 cells incubated with GO–FA/Ce6 for 8 h before or after FR saturation with free FA. In green, membranes stained with CellMask (excitation channel: 488 nm, emission: 500–550 nm); in red, GO–FA/Ce6.

To prove the FR-mediated internalization, we performed a competition assay where the pretreatment of MCF-7 cells with FA led to a reduced cellular uptake of GO–FA/Ce6, confirming the FR-mediated endocytosis (Figure 5). These results are also consistent with previous reports [51,54].

### 3.4. Combined Effect of PTT and PDT by GO–FA/Ce6 on MCF-7 Cells

**Figure 6:**
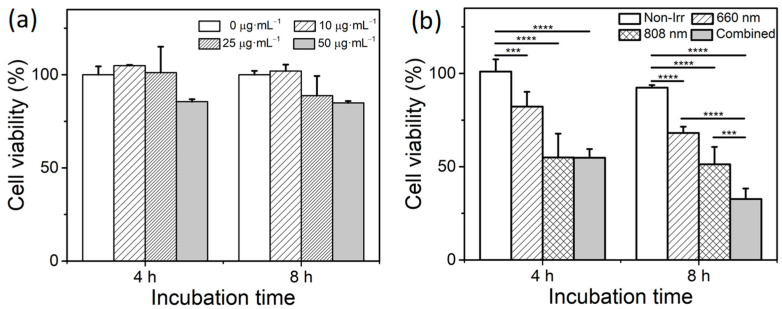
(**a**) Cytotoxicity of GO–FA/Ce6 on MCF-7 cells and (**b**) comparison of MCF-7 viability following PTT, PDT and combined phototherapy after the incubation with GO-FA/Ce6 (at 25 μg·mL^−1^) for 4 and 8 h. MCF-7 cells incubated with GO–FA/Ce6 (at 25 μg·mL^−1^) for 4 and 8 h (Non-Irr) were used as control. The results are expressed as the means ± SD (*n* ≥ 3). The statistical analyses were performed by the Student’s *t*-test. * *p* < 0.05; ** *p* < 0.01; *** *p* < 0.001; **** *p* < 0.0001 compared with two groups.

The cytotoxicity of GO–FA/Ce6 on MCF-7 cells was also evaluated after incubation for 4 and 8 h using the MTS assay (Figure 6a). The double-functional GO showed only a slight, nonsignificant decrease in the cell viability of MCF-7 cells. After 4 h incubation, the GO conjugate presented no cytotoxicity at the concentrations of 10 and 25 μg·mL^−1^ with a cell viability around 100%, while a cell viability of 85% was measured when the concentration increased to 50 μg·mL^−1^. At 25 μg·mL^−1^, the cell viability decreased only to 89%. As the standard deviation was rather high, the difference in percentage at the two time points was not statistically significant. At 50 µg·mL^−1^, we observed no difference in cell viability between 4 and 8 h.

To study the photo-induced cytotoxicity of GO–FA/Ce6 on MCF-7 cells, the cell viability was evaluated by the LIVE/DEAD^®^ assay after irradiation at 660 nm and 808 nm in the presence of GO–FA/Ce6 at 25 μg·mL^−1^ (Figure 6b). The LIVE/DEAD^®^ viability/cytotoxicity assay is a two-color fluorescence test based on the simultaneous determination of live and dead cells with two probes measuring the intracellular esterase activity (calcein AM) and plasma membrane integrity (ethidium homodimer, EthD-1). The cells were incubated with GO–FA/Ce6 for 4 and 8 h before the cell medium was replaced with 200 μL of fresh cell culture medium. The laser irradiation without nanomaterials showed a minimal decrease in the cell viability on MCF-7 cells (Figure S4). In contrast, the 808 nm irradiation of the cells incubated with GO–FA/Ce6 induced a high killing efficiency of the MCF-7 cells, with a cell viability decrease to 54% (after 4 h incubation) and to 51% (after 8 h incubation). The photothermal effect not only directly kills the cancer cells, but hyperthermia would also sensitize cells leading to a higher therapeutic effect of the PDT treatment [17,55].

The photodynamic treatment exhibited an incubation time-dependent killing efficiency. After 4 h incubation followed by the 660 nm irradiation, the cell viability decreased to 82% compared to the nonirradiated group (~100%) (Figure 6b). A lower viability (68%) was observed in the group with longer incubation time, revealing a higher PDT efficiency. To study the combined effect, the cytotoxicity of MCF-7 cells after the combined PTT and PDT treatment using GO–FA/Ce6 was assessed (Figure 6b). For this purpose, the cells were treated with an 808 nm laser irradiation for PTT followed by 660 nm irradiation for PDT. Although we observed no combined effect for the cells after 4 h incubation, 67% of the MCF-7 cells were killed after 8 h incubation, which was much higher than the photothermal treatment alone (49% of cell death) and photodynamic treatment (32% of cell death). The higher photocytotoxicity after the combined treatment at 8 h incubation compared to 4 h incubation could come from a higher accumulation of the conjugate in the MCF-7 cells. This result reveals that a combined effect was achieved by using two laser wavelengths in the presence of GO–FA/Ce6.

**Figure 7:**
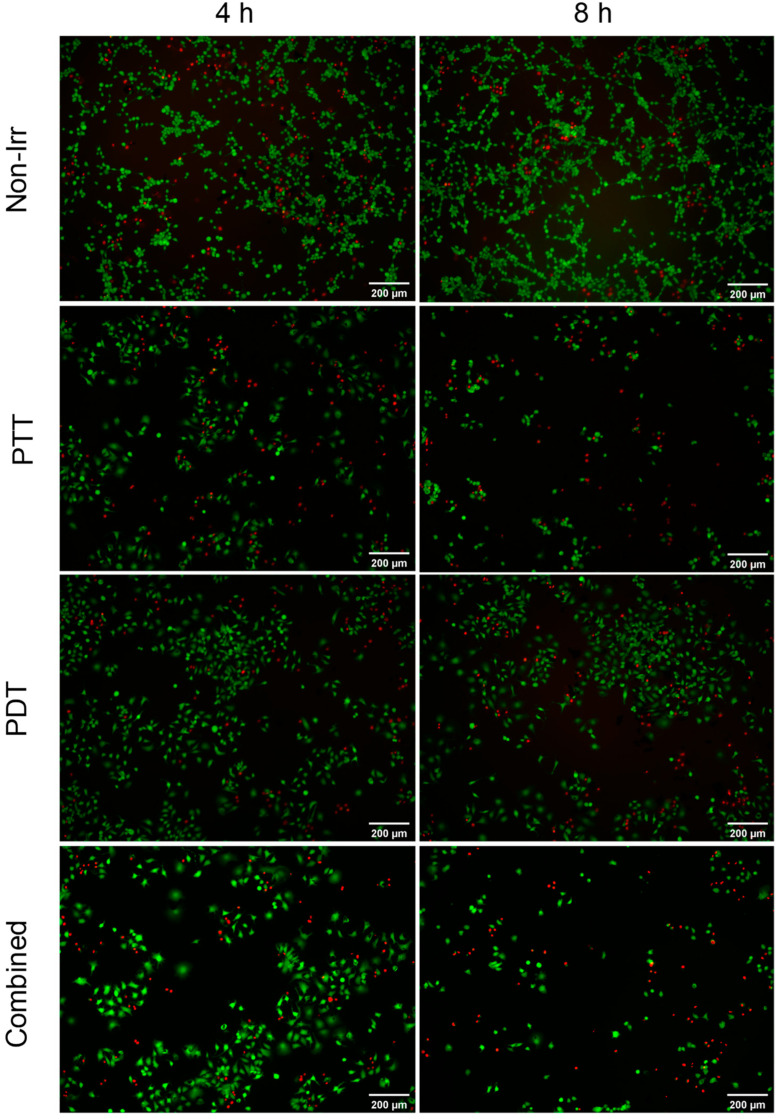
Fluorescence images of calcein AM (green signal from live cells, E_x_ 495 nm and E_m_ 517 nm) and EthD-1 (red signal from dead cells, E_x_ 528 nm and E_m_ 617 nm) double-stained cells after PTT, PDT and combined phototherapy treatments following the incubation with GO–FA/Ce6 for 4 and 8 h. MCF-7 cells incubated with GO–FA/Ce6 for 4 and 8 h without irradiation (Non-Irr) were used as control.

Following the quantification of viable cells by the LIVE/DEAD^®^ test, fluorescence microscopy also confirmed the change of live and dead cell numbers according to the different treatments (Figure 7). Compared to the control group, the images of all treated cells showed the presence of a lower number of cells. The combined laser-irradiated cells after 8 h incubation were far fewer in comparison to the other treated groups, which was consistent with the cell viability experiment.

Although it is difficult to make a comparison among the different systems used for the evaluation of antitumor efficiency using various nanomaterials, the covalently functionalized GO–FA/Ce6 showed a higher photocytotoxicity at a lower dose compared to other similar studies using GO–Ce6-based conjugates [29,56,57]. For example, our GO–FA/Ce6 showed a combined PTT and PDT anticancer efficiency on MCF-7 cells comparable to the cucurbit[7]uril–NGO–Ce6–oxaliplatin–hyaluronic acid nanocomplex (CNGH^OX^) on murine melanoma B16 cells [56]. However, the amount of Ce6 was much higher in CNGH^OX^ (5 μg·mL^−1^ vs. 45 ng·mL^−1^ in our GO–FA/Ce6), alerting to the fact that side effects of chemotherapy should be considered. In addition, several articles have demonstrated the combined effect of PTT/PDT by loading Ce6 on GO through physisorption [29,58]. However, the nonspecific release of the adsorbed Ce6 from the GO surface should be considered in this case, especially in a biological environment rather than in PBS [59]. Even if noncovalent functionalization strategies could lead to a higher loading efficiency up to 80% (m_Ce6_/m_GO_) [29], the Ce6 molecules might be removed after excessive washing. We demonstrated that the final loading efficiency was lower for Ce6 adsorbed on GO compared to the covalent conjugation (Figure S2). Comparatively, we obtained a similar or higher therapeutic efficiency while using a lower dose of conjugate (based on Ce6 equivalent.) [56,60]. Additionally, an inappropriate covalent functionalization may also lead to lower phototherapeutic efficiency. As an example, the carboxylation of GO is reported as one strategy to increase the amount of carboxylic acids on GO, which could be further derivatized via amidation with amines [61]. However, the excessive use of a base to perform the carboxylation reaction can lead to the deoxygenation of GO, resulting in a lower loading efficiency [48]. This might be a reason for the application of a high dose of GO–Ce6 conjugates in some studies to achieve sufficient antitumoral efficiency [62].

### 3.5. PTT and PDT of GO–FA/Ce6 on Macrophages

As the GO–FA/Ce6 conjugate has shown a good potential in the treatment of cancer cells by combining PTT and PDT to achieve a synergetic effect, we extended the use of this nanomaterial to RAW 264.7 macrophages for the potential treatment of cancer [63,64] and inflammatory diseases [65]. Tumor-associated macrophages can exhibit tumoricidal M1 or regenerative M2 phenotypic activation states. M2 tumor-associated macrophages are immunosuppressed and promote tumor invasion [38]. Thus, combining immunotherapy with other therapeutic approaches is a promising strategy against cancer [66].

#### 3.5.1. Cytotoxicity and Cellular Uptake of GO–FA/Ce6 in Macrophages

**Figure 8:**
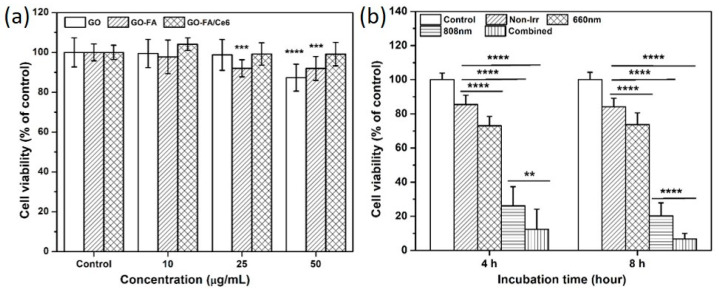
(**a**) Cytotoxicity of GO, GO–FA and GO–FA/Ce6 on RAW 264.7 macrophages after 24 h incubation and (**b**) comparison of RAW 264.7 cell viability following PTT, PDT and combined phototherapy after incubation with GO–FA/Ce6 for 4 and 8 h. Untreated RAW 264.7 macrophages were used as control. The results are expressed as the means ± SD (*n* ≥ 3). The statistical analyses were performed by the Student’s *t*-test. * *p* < 0.05; ** *p* < 0.01; *** *p* < 0.001; **** *p* < 0.0001 compared with two groups.

We first evaluated the cytotoxicity of GO–FA/Ce6 on RAW 264.7 macrophages. The cytotoxicity of GO–FA/Ce6 was evaluated at different concentrations after incubation for 24 h with the MTS assay (Figure 8a). Overall, the three materials resulted in being relatively safe on RAW 264.7 macrophages, even at the highest concentration (50 μg·mL^−1^). The cell viability decreased to about 90% after exposure to GO or GO–FA at 50 μg·mL^−1^. The double-functional GO showed no effects on RAW 264.7 macrophages at all concentrations, indicating that the RAW 264.7 macrophages have a good tolerance against GO–FA/Ce6.

**Figure 9:**
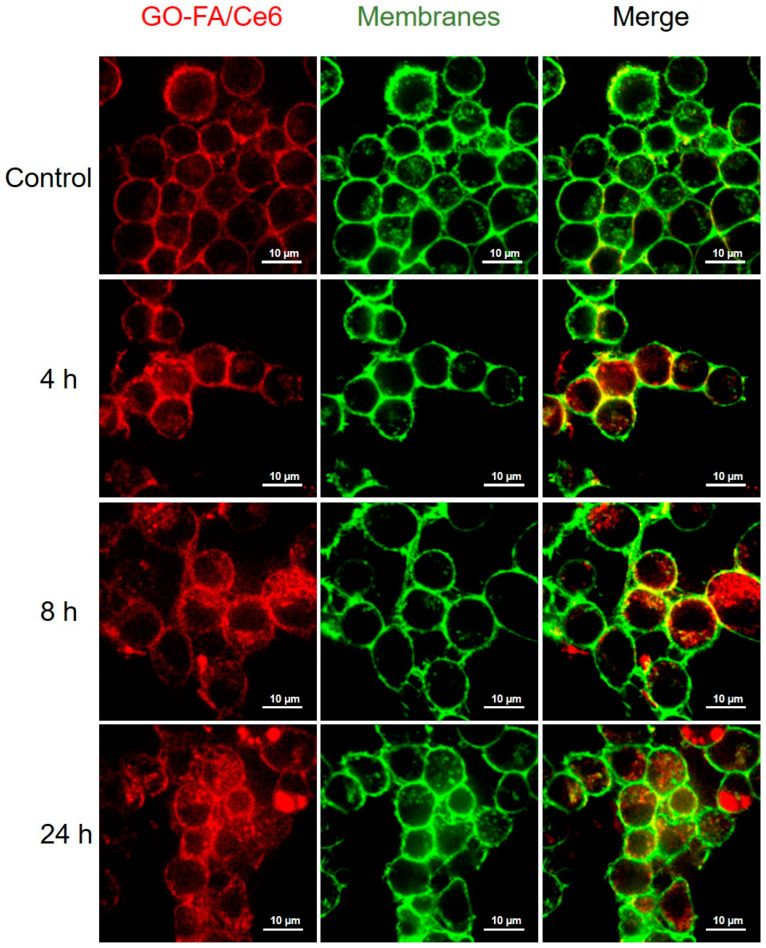
Confocal microscopy images of RAW 264.7 macrophages incubated with 25 μg·mL^−1^ of GO–FA/Ce6 at different time points. The untreated RAW 264.7 macrophages were used as control. In green, membranes stained with CellMask (excitation channel: 488 nm, emission: 500–550 nm); in red, GO–FA/Ce6.

The cellular uptake of GO–FA/Ce6 by RAW 264.7 macrophages was then evaluated using confocal microscopy by tracking the fluorescence of Ce6 on GO at different time points (0, 4, 8 and 24 h) (Figure 9). CellMask green was applied to visualize the cell membranes. As shown in Figure 9, GO–FA/Ce6 was internalized by the RAW 264.7 macrophages already after 4 h, displaying a remarkable red emission. After 8 h incubation, the cytoplasm of the cells was full of red dots. Since we observed a higher internalization at short times (4 and 8 h), we decided to perform the photothermal and photodynamic experiments at these incubation time points with the GO–FA/Ce6 at the concentration of 25 μg·mL^−1^, just as for the MCF-7 cells.

#### 3.5.2. Phototoxicity of GO–FA/Ce6 on Macrophages

To study the photo-induced cytotoxic effect of GO–FA/Ce6 on RAW 264.7 macrophages, the cell viability was evaluated by the LIVE/DEAD^®^ assay after irradiation using 660 nm and 808 nm lasers in the presence of GO–FA/Ce6 (Figure 8b). Compared to the results of cytotoxicity assessed by MTS, the LIVE/DEAD^®^ assay evidenced a cell viability at around 85% in the group without irradiation, both after 4 h and 8 h incubation. After applying the 660 nm and 808 nm irradiation, a clear photo-triggered cytotoxicity was measured (Figure 8b). The 808 nm light induced a high killing effect by GO–FA/Ce6 on RAW 264.7 macrophages, significantly decreasing the percentage of live cells to 25% (after 4 h incubation) and to 20% (after 8 h incubation), revealing that the macrophages were more sensitive to the hyperthermia induced by the photothermal effect of GO–FA/Ce6 than MCF-7 cells. The efficiency of the photodynamic treatment on RAW 264.7 macrophages was lower than that observed by PTT. After the irradiation with 660 nm laser, the cell viability decreased to 72%. The low killing efficiency of PDT in RAW cells was probably due to the high resistance of the macrophages to ROS. The protective mechanisms against the oxidative burst during the inflammatory process was acquired during differentiation from monocytes through several antioxidant and cytoprotective pathways. These antioxidant and cytoprotective processes can help macrophages to reduce the oxidative burden, as well as the use of ascorbate (vitamin C) and α-tocopherol (vitamin E) [67].

To perform the combined PTT/PDT treatment, the cells were incubated with GO–FA/Ce6 for 4 and 8 h before the cell medium was replaced with 200 μL of fresh cell culture medium. The cells were then treated with an 808 nm laser irradiation for PTT followed by 660 nm irradiation for PDT. In both groups, a clear combined effect was observed. After 4 h incubation, 87% of the macrophages were killed by the two laser-induced cytotoxic effects, much higher than the photothermal treatment alone (75% of cell death) and photodynamic treatment (only 28% of cell death). When the incubation time was prolonged to 8 h, an even higher photocytotoxicity was obtained (94% of cell death). The higher combined photocytotoxicity can be explained by a higher accumulation of the materials inside the macrophages over time, which is consistent with the results obtained using the MCF-7 cells. Endowed with good biocompatibility, GO–FA/Ce6 showed a sufficient killing efficiency of the macrophages, making this material a conjugate with a higher potential for the treatment of inflammatory diseases or cancer [68].

**Figure 10:**
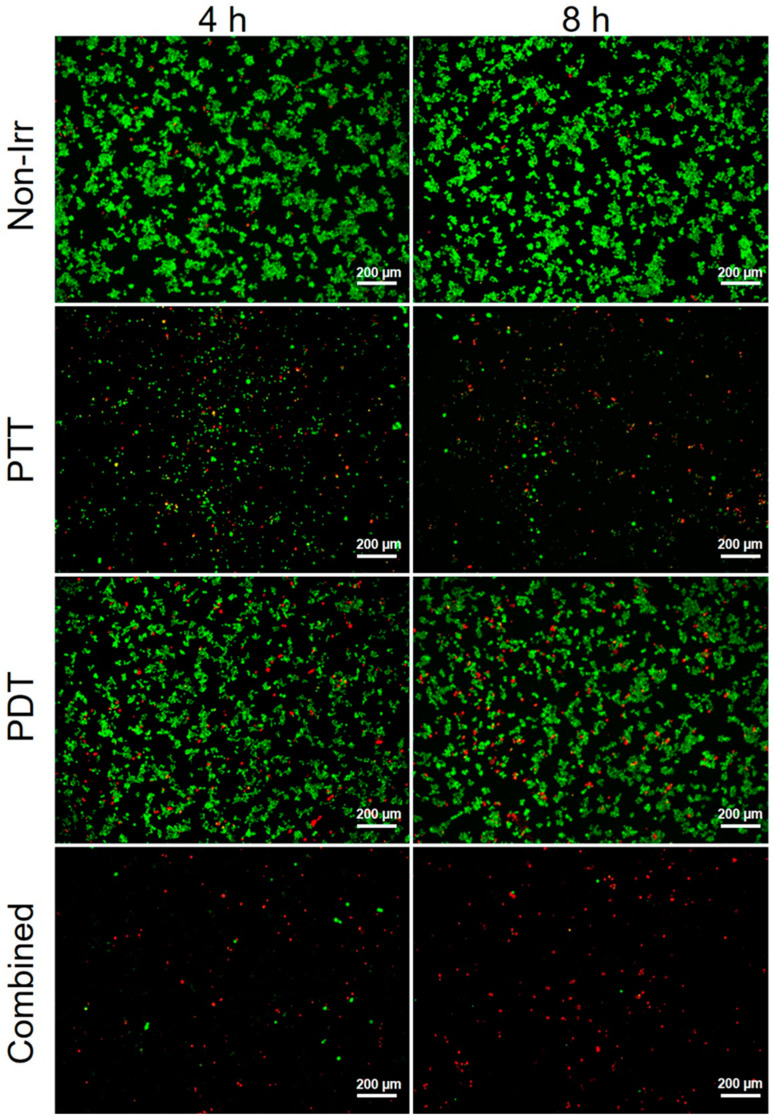
Fluorescence images of calcein AM (green signal from live cells, E_x_ 495 nm and E_m_ 517 nm) and EthD-1 (red signal from dead cells, E_x_ 528 nm and E_m_ 617 nm) double-stained RAW 264.7 macrophages after PTT, PDT and combined phototherapy treatments following the incubation with GO–FA/Ce6 for 4 and 8 h. RAW 264.7 macrophages incubated with GO–FA/Ce6 for 4 and 8 h without irradiation (Non-Irr) were used as control.

The fluorescence staining also proved the high killing efficiency of GO-FA/Ce6 on macrophages after irradiation (Figure 10). Compared to the control group and the nonirradiated group, the images of the cells treated by PTT/PDT displayed fewer cells. The 808 nm laser-irradiated cells were far fewer in comparison to the PDT and control groups, which was consistent with the results of the cell viability experiment. The 660 nm laser-irradiated groups showed a higher cell number compared to the 808 nm laser-irradiated groups, but still lower than the nonirradiated group. When we applied both PTT and PDT to the RAW cells, the combined effect was also observed. There were far fewer cells alive in the group of 4 h incubation, while almost all the cells were dead after 8 h incubation following the combined laser irradiation (Figure 10).

#### 3.5.3. Cytokine Expression after GO–FA/Ce6 Treatment

Macrophages can contribute to tumor growth and progression by promoting tumor cell proliferation and invasion, fostering tumor angiogenesis and suppressing antitumor immune cells. In order to explore the activation profile of the macrophages following their incubation with the three materials, we determined the amount of the proinflammatory cytokines IL6, TNFα and IL-1β in the culture supernatants by ELISA. As shown in Figure S5, the release of proinflammatory cytokine IL6 was not modified by incubation of RAW 264.7 macrophages with GO, GO–FA and GO–FA/Ce6 at all doses tested in comparison to the untreated control. The TNFα production induced by GO, GO–FA and GO–FA/Ce6 was similar, even if there was a very slight increase in high concentrations (50 μg·mL^−1^) of GO–FA and GO–FA/Ce6, but this small increase was almost negligible, compared with the untreated control. However, we observed an increase in IL-1β secretion after the incubation of the macrophages with GO–FA and GO–FA/Ce6. This pro-inflammatory cytokine was produced by tumoricidal M1 macrophages, and it is reported that IL-1β is able to inhibit the growth of the primary tumor by impairing the infiltration of innate immune cell subsets with potential anticancer functions, which would help to improve the combined phototherapy for cancer treatment [69].

## 4. Conclusions

A FA/Ce6 double-functionalized GO was successfully prepared and used for in vitro PTT/PDT combined therapy on MCF-7 cells and RAW 264.7 macrophages. GO–FA/Ce6 exhibited good photothermal properties and a high ROS generation capacity. The nanomaterial was able to penetrate rapidly into cancer cells due to FR-mediated endocytosis and into macrophages. A higher therapeutic efficiency to kill cancer cells was achieved by combining photothermal and photodynamic therapy compared to the single treatments. GO–FA/Ce6 also showed the capacity to efficiently eliminate RAW 264.7 macrophages by PTT/PDT, with a killing efficiency of up to 94% being achieved. This type of cell showed a higher sensitivity to PTT compared to the cancer cells. These results prove the promising therapeutic effect of combined PDT and PTT for anticancer and anti-inflammatory therapy using a multifunctional GO. Moreover, our strategy can be easily extended to the preparation of various covalently multifunctionalized GO by changing the type of functionalities with different targeting ligands and therapeutic molecules for disease treatments. This method can be performed in water or organic solvents without catalyst or heating, which is appropriate for the conjugation of pH- or temperature-sensitive moieties. Furthermore, the covalent conjugates are more stable than noncovalent complexes obtained by physisorption, which is crucial for the application in targeted disease treatments. To explore further the potential application of our GO–FA/Ce6 conjugate in cancer therapy, the combined PTT and PDT will be translated into an in vivo model to evaluate the tissue distribution and tumor eradication in a future study.

## Data Availability

Data are available from the authors upon request.

## Supplementary Materials

The following supporting information can be downloaded at: https://www.mdpi.com/article/10.3390/pharmaceutics14071365/s1, Figure S1: Standard curve of Ce6; Figure S2: (a) UV-Vis spectra of GO–FA/Ce6 and GO–FA/Ce6–CTR. The Ce6 loaded on GO–FA via physisorption was calculated as 0.9 μmol·g^−1^; Figure S3: Thermogravimetric analysis of GO and GO–FA/Ce6 performed under N2 atmosphere; Figure S4: Phototoxicity of 808 nm (2 W∙cm^−2^, 10 min) and 660 nm (0.2 W∙cm^−2^, 10 min) laser irradiation on MCF-7 cells; Figure S5: Cytokine production by RAW 264.7 macrophages.
