# Supplementary material for: Combined Photothermal and Photodynamic Therapy for Cancer Treatment Using a Multifunctional Graphene Oxide
Source: Pharmaceutics. 2022 Jun 28;14(7):1365. doi: 10.3390/pharmaceutics14071365 (PMC9318106; doi:10.3390/pharmaceutics14071365)
Supplement: Supplementary file 1 [file pharmaceutics-14-01365-s001.zip › pharmaceutics-1737637-supplementary.pdf]

Supplementary Materials:

Combined Photothermal and Photodynamic Therapy for Cancer Treatment Using a Multifunctional Graphene Oxide

Shi Guo,[1,&] Zhengmei Song,[1,&] Ding-Kun Ji,[1,#] Giacomo Reina,[1,$] Jean-Daniel Fauny,[1] Yuta Nishina,[2] Cécilia Ménard-Moyon,[1] Alberto Bianco [1,*]

1. Experiment Section

1.1 Materials and methods

All the chemicals and solvents were obtained from commercial suppliers and used without purification. *O*-(2-aminoethyl)-*O'*-[2-(Boc-amino)ethyl]decaethylene glycol (BocNH-PEG_{10}-NH_2) was purchased from Polypure AS. Folic acid was purchased from ACROS. 1-Ethyl-3-(3-dimethylaminopropyl)carbodiimide hydrochloride (EDC·HCl) and *N*-hydroxysuccinimide (NHS) were purchased from Alfa Aesar. Dihydrorhodamine-123 (DHR123) purchased from Sigma-Aldrich. The solvents used during the reaction were analytical grade. Water was purified by a Millipore filter system MilliQ®. For dialysis, MWCO 12,000-14,000 Da membranes were purchased from Spectrum Laboratories, Inc.

1.2 Instruments for the characterization of materials

High-performance liquid chromatography analyses were performed on a Waters Alliance e2695 instrument, with integrated autosampler and a Waters 2998 PDA detector, using a Macherey–Nagel Nucleosil 100–5 C 18 column 4.6×125 mm (gradient: 0-100% of A and 100-0% of B in 20 min at 1.2 mL·min^{-1} flow rate). Eluants for HPLC: A = H_2O+0.1% TFA, and B = MeCN+0.08% TFA. Fluorescence measurements were performed in a water suspension at a concentration of 10 μg·mL^{-1} with a Jasco FP8003 fluorimeter using a swig xenon 450 W lamp and were corrected for the baseline and the solvent. The excitation wavelength was 420 nm and the emission range was measured between 520 nm and 800 nm. UV-Vis absorption spectra were recorded on a Cary 5000 UV-Vis-NIR spectrophotometer using 1 cm path quartz glass cuvettes and were corrected for the baseline and the solvent. The temperature was controlled at 25℃ using a Peltier system. Transmission electron microscopy analysis was performed on a Hitachi H600 with an accelerating voltage of 75 kV. The samples were dispersed in water/ethanol (1:1) at a concentration of 16 μg·mL^{-1} and the suspensions were sonicated for 10 min. Ten microliters of the suspensions were drop-casted onto a copper grid (Formvar film 300 Mesh, Cu from Electron Microscopy Sciences) and left for evaporation under ambient conditions. Thermogravimetric analysis was performed on a TGA1 (Mettler Toledo) apparatus from 30°C to 900°C with a ramp of 10°C·min^{-1} under N_2 atmosphere with a flow rate of 50 mL·min^{-1} and platinum pans. LC/MS analyses were performed on a LC/MS instrument equipped with a Thermo Scientific VANQVISH Flex UHPLC (Hypers II GOLD column, 50×2.1 mm, 1.9 μm) integrated with a Thermo Scientific LCQ Fleet ion-trap. Deconvolution of the data was performed in MagTran 1.03 (Amgen, Thousand Oaks, CA).

1.3 Immunostaining

A suspension of GO-FA/Ce6 was deposited on a TEM grid. The grid was first verified by TEM that the functionalized carbon nanomaterials were well dispersed. This grid was then re-hydrated by deposition of the grid on a drop of water (300 μL) for 15 min. The grid was then incubated in 40 μL acetylated BSA (1% in PBS) for 45 min. Then the grid was deposited on a 40 μL drop of anti-FA antibody [using mouse monoclonal folic acid antibody (FA1) stock solution (7 mg·mL^{-1}), ref. NB100-72975, from NOVUS BIOLOGICALS, LLC] and incubated for 2 h (dilution 1/50 with 0.1% acetylated BSA in PBS). The antibody was removed and washed with PBS (4 times for 10 min each time) and with

water (4 times for 10 min each time) by deposition of the grid on 300 μL of PBS or water. Incubation of the grid with the secondary antibody (using anti-mouse antibody coupled to 10-nm gold nanoparticles, ref. GAM 10 nm AURION 810.177, from AURION) for 2 h (dilution 1/50 in 0.1% acetylated BSA in PBS, centrifuging the antibody 2 min at 10000 g before dilution) was performed by deposition of the grid on a 40 μL drop. The grid was washed with PBS (3 times for 10 min each time) and with water (3 times for 10 min each time) by dropping the grid on 300 μL of PBS or water. Two control samples were also used, using GO without FA (GO-FA-CTR) followed by the incubation with the anti-FA antibody and the secondary antibody, and using hRANK-M331 antibody (mouse IgG1monoclonal antibody against human RANK-M331 from Immunex Corporation, dilution 1/20 in 0.1% acetylated BSA in PBS) instead of the anti-FA antibody on GO-FA followed by the incubation with the secondary antibody.

1.4 Photothermal experiments

Dispersions of GO-FA/Ce6 in water at different concentrations were prepared using sonication. The photothermal conversion of the suspensions was examined by monitoring the temperature increase during irradiation using an 808-nm laser (MRL-III-808-2W from Changchun new industries optoelectronics tech.co., ltd) at a power density of 2 $W \cdot cm^{-2}$ from 0 to 15 min. The distance between the laser and the sample was kept constant at 3 cm. Each photothermal experiment was repeated three times. The temperature was monitored using an infrared thermal imaging camera by recording the maximum temperatures in the images.

1.5 ROS generation assay

DHR123 was used to evaluate ROS generation in water solutions. Light-triggered generation of ROS oxidizes DHR123 to fluorescent rhodamine 123. In a typical assay, a suspension of GO-FA/Ce6 (10 $\mu g \cdot mL^{-1}$) was prepared in water and DHR123 (final concentration at 20 nM) was added. Then, the mixture was irradiated under 660 nm laser (MRL-III-660D-300mW from Changchun new industries optoelectronics tech.co., ltd; 0.2 $W \cdot cm^{-2}$) for 10 min and the emission intensity in the range from 495 nm to 900 nm was recorded with excitation at 485 nm.

1.6 Cell Culture

Human Caucasian breast adenocarcinoma (MCF-7) and RAW 264.7 macrophages were cultured in high-glucose Dulbecco's modified Eagle medium (DMEM) with 10 $\mu g \cdot mL^{-1}$ gentamycin (Lonza BioWhittaker), 10 mM *N*-(2-hydroxyethyl)-piperazine-*N*′-ethanesulfonic acid (Lonza BioWhittaker), 0.05 mM β-mercaptoethanol (Lonza BioWhittaker) and 10% fetal bovine serum (FBS).

1.7 Cell viability assay

MTS assay and LIVE/DEAD® assay were performed to measure the cell viability according to the manufacturer's instructions. For MTS assay, MCF-7 and RAW 264.7 cells were seeded in a 96-well plate (Greiner bio-one, Germany) at a density of 6×10^3 cells/well and 1×10^4, respectively. The cells after the treatment with GO were incubated with MTS solution in cell culture media (10% v/v solution) for 60 min at 37°C. UV-Vis absorption was measured at 490 nm under the Microplate Reader (Thermo, Varioskan Flash), and MTS in the cell culture medium alone was included as blank. The experiments were performed with at least three replicates. The results were expressed as percentage cell viability versus control. For LIVE/DEAD® assay, a solution A (2 μM calcein AM), solution B (4 μM EthD-1) and a working solution (2 μM calcein AM and 4 μM EthD-1) were freshly prepared before adding to the cells. MCF-7 and RAW 264.7 cells after the treatment with GO were incubated with the working solution for 30 min at room temperature. Three control samples were also performed using solution A, solution B and the working solution. Fluorescence was measured at the respective excitation and emission wavelength of 480 nm and 571 nm for calcein AM and 528 nm and 671 nm for EthD-1, using the Microplate

Reader (Thermo, Varioskan Flash). The cell viability was calculated according to the manufacturer's instructions.

1.8 Confocal imaging

Confocal images were obtained with a Zeiss Axio Observer Z1 spinning disk confocal microscope equipped with a 63 or 100 × oil objective. The fluorescence signal from CellMask (Sigma-Aldrich) was obtained using a 488-nm laser excitation and recording in the green channel (BP525/50), whereas GO-FA/Ce6 were recorded using a 405-nm laser excitation in the far-red (FR) channel (BP690/50). The images were then treated with ImageJ software. To confirm the receptor-mediated uptake, competition experiments were conducted where the cell culture was pre-treated with 50 μL of saturated FA solution for 1.5 h prior to GO-FA/Ce6 treatment. The saturated FA solution was prepared according to the following procedure: 50 mg of FA was added to 1 mL of PBS and the suspension was sonicated for 10 min. After centrifugation at 10000 rpm for 20 min, the supernatant was collected.

1.9 PDT and PTT experiments

MCF-7 cells (6.0×10^3/well) or RAW 264.7 (1.0×10^4/well) were seeded on a 96-well microplate for 24 h. The cells were incubated with GO-FA/Ce6 for 4 or 8 h the cell medium was replaced with 200 μL of fresh cell culture medium. Samples with a total volume of 100 μL of cell culture media in 96-well plates were irradiated for 10 min with a 660-nm laser (0.2 $W\cdot cm^{-2}$) for PDT or with a 808-nm laser (2 $W\cdot cm^{-2}$) for PTT. After 24 h incubation, the culture media were removed and the cell viability was measured using different assays.

1.10 Combined PDT and PTT experiments

MCF-7 cells (6.0×10^3/well) were seeded on a 96-well microplate for 24 h. The cells were incubated with GO-FA/Ce6 for 4 or 8 h the cell medium was replaced with 200 μL of fresh cell culture medium. Samples with a total volume of 100 μL of cell culture media in 96-well plates were irradiated with an 808-nm laser (2 $W\cdot cm^{-2}$) for PTT for 10 min followed by a 660-nm laser (0.2 $W\cdot cm^{-2}$) irradiation for PDT. After 24 h incubation, the culture media were removed and the cell viability was measured using LIVE/DEAD® assay.

1.11 Fluorescence microscopy imaging

The cells treated with light irradiation were stained with LIVE/DEAD® kit following the same protocol described in the cell viability part. The images were taken by fluorescence microscopy (ZEISS, Axiovert 200 M) at 5× magnification.

1.12 Cytokine assay

RAW 264.7 macrophages were seeded in 96-well plates at a density of 1×10^4 cells per well. After 24 h, the nanomaterials with different concentrations (10, 25, 50 $\mu g\cdot mL^{-1}$) were added to the macrophages for 24 h, the cells cultured in the medium without nanomaterials were used as control. The cells were treated with 1 $\mu g\cdot mL^{-1}$ of lipopolysaccharide (LPS) for 24 h as positive control. The supernatants were collected, and the concentrations of IL6 and TNFα were determined using ELISA kits (BD Biosciences), respectively, following the manufacturer's instructions.

1.13 Statistical analysis

The results are presented as mean ± standard deviation of at least three independent experiments. The numbers of samples per group in each experiment are indicated in the corresponding figure legends as "n". Differences between groups were evaluated with the Student's t-test for two groups. *, **, *** and ****denote the p-values less than 0.05, 0.01, 0.001 and 0.0001, respectively.

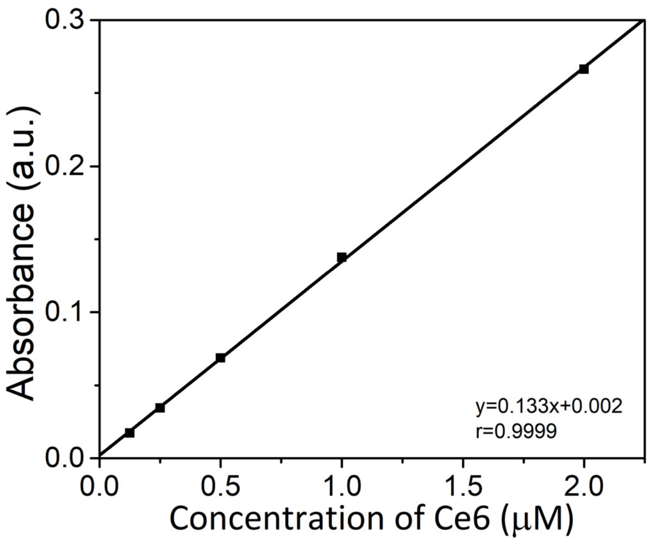

Figure S1. Standard curve of Ce6 by measuring the Soret band's absorption around 420 nm in water.

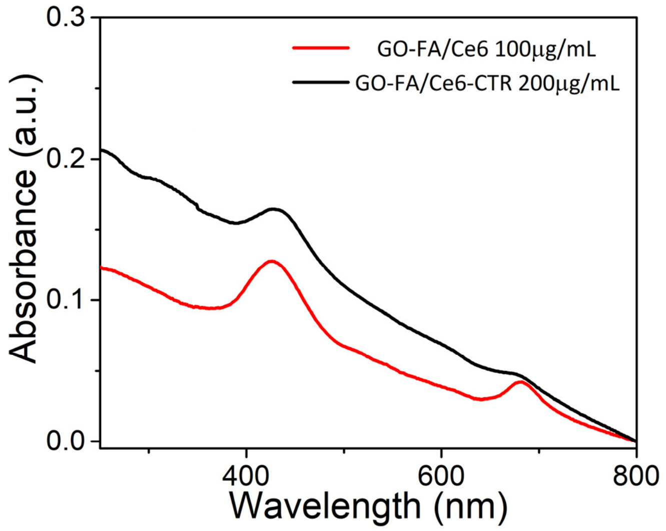

Figure S2. UV-Vis spectra of GO-FA/Ce6 and GO-FA/Ce6-CTR in water. The Ce6 loaded on GO-FA via physisorption was calculated as 0.9 μmol·g^{-1}.

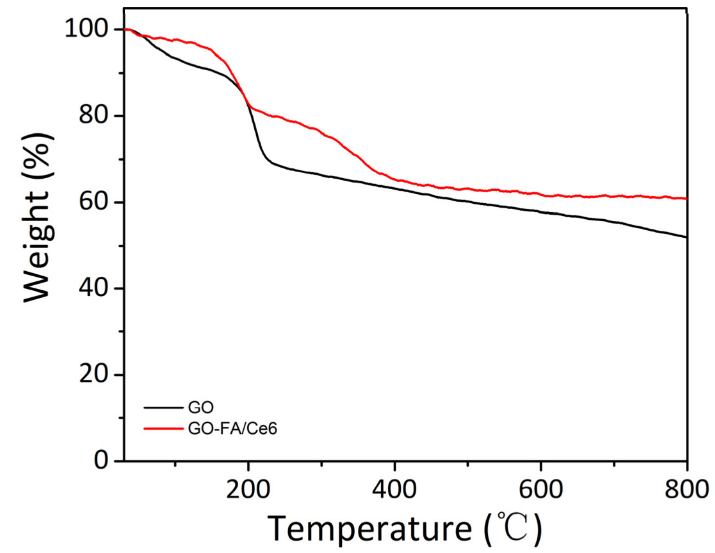

Figure S3. Thermogravimetric analysis of GO and GO-FA/Ce6 performed under N_2 atmosphere.

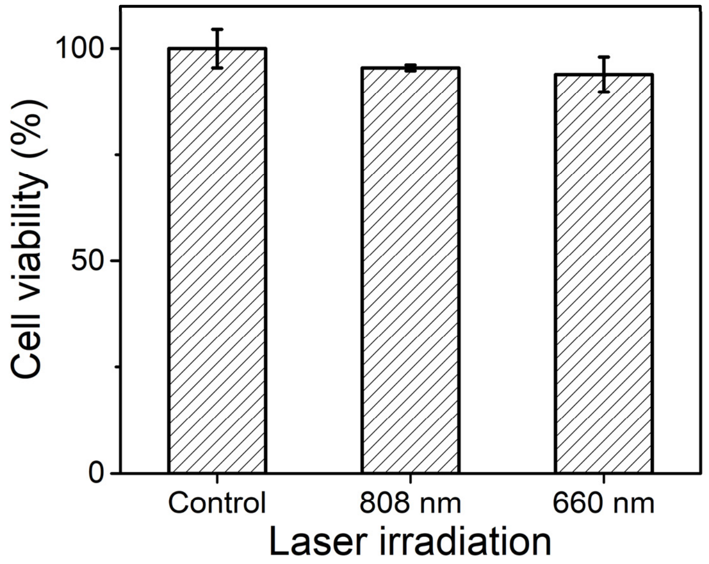

Figure S4. Phototoxicity of 808-nm (2 W·cm^{-2}, 10 min) and 660-nm (0.2 W·cm^{-2}, 10 min) laser irradiation on MCF-7 cells. Untreated MCF-7 cells were used as control.

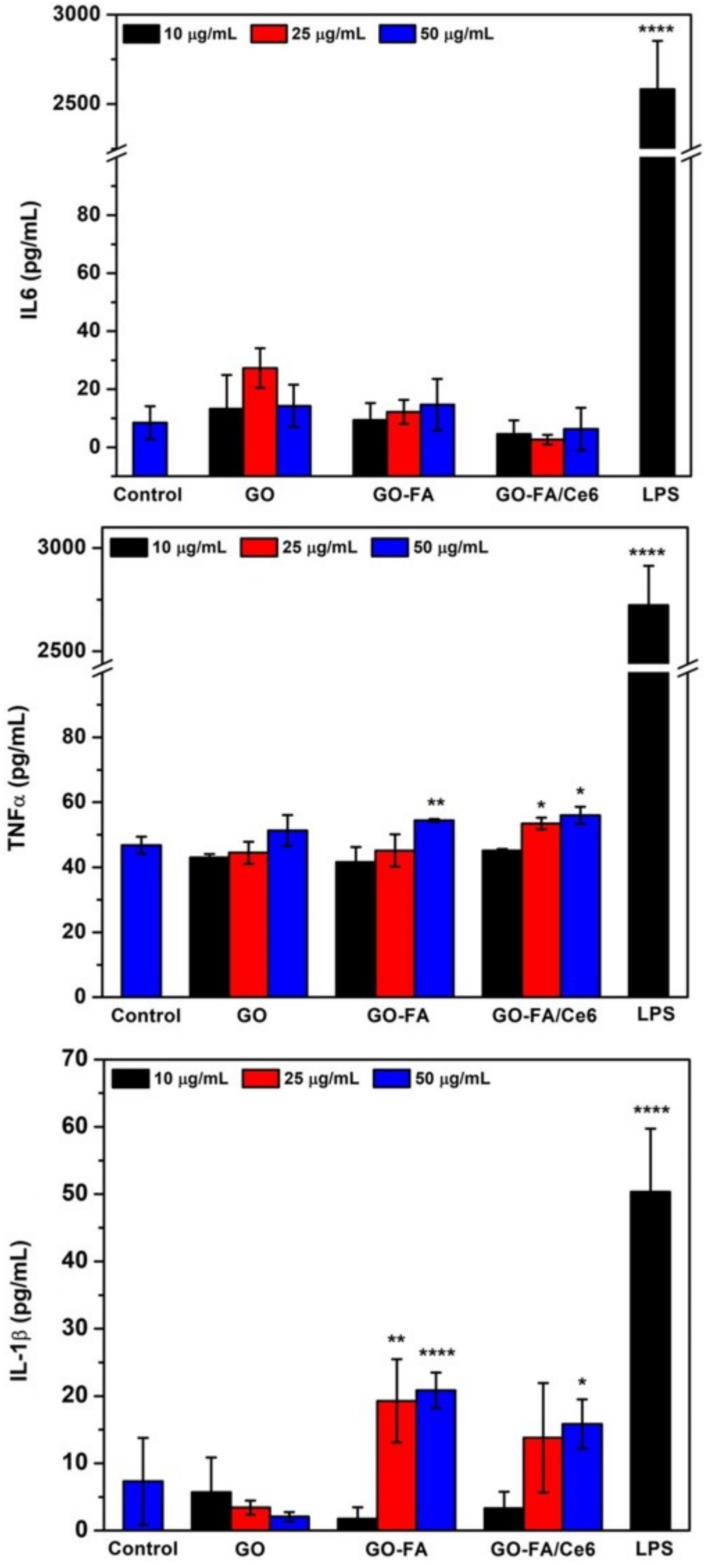

Figure S5. Cytokine production by RAW 264.7 macrophages. IL6, TNFα and IL-1β levels were determined after incubation at increasing concentrations of GO, GO-FA and GO-FA/Ce6. The results are expressed as the means ± SD (n=3). The statistical analysis were performed by the Student's *t*-test. p < 0.05; **p < 0.01; ***p < 0.001, ****p < 0.0001 compared with the untreated control.
